# Impact of Calcium–Magnesium Ratio in Purified Water Remineralization on Calcium Oxalate Crystal Formation and Renal Injury

**DOI:** 10.3390/nu18050792

**Published:** 2026-02-27

**Authors:** Yingbin Zhang, Jiaohua Luo, Yao Tan, Zhiqiang Wang, Kun Qian, Weiyan Chen, Ke Cui, Ji-An Chen, Yujing Huang

**Affiliations:** 1 Department of Health Education, College of Preventive Medicine, Army Medical University, Chongqing 400038, China; yingbinzhang@tmmu.edu.cn; 2 Department of Environmental Hygiene, College of Preventive Medicine, Army Medical University, Chongqing 400038, China; ljh978@tmmu.edu.cn (J.L.); xiaoyue7122@tmmu.edu.cn (Y.T.); weiyanchen@tmmu.edu.cn (W.C.); cuike@tmmu.edu.cn (K.C.); 3 Department of Environmental Science and Engineering, School of Environmental Studies, China University of Geosciences, Wuhan 430074, China; cugwangzq@126.com (Z.W.); qkwell2046@cug.edu.cn (K.Q.)

**Keywords:** urinary stone, renal injury, calcium, magnesium, purified water, remineralization

## Abstract

Despite the known association between calcium and magnesium in drinking water and stone risk, the difference in stone prevention of purified water remineralized with varying calcium-to-magnesium ratios (Ca:Mg) remains unclear. **Objectives**: This study investigates the impact of different Ca:Mg in the remineralization of purified water on calcium oxalate crystallization and renal injury. **Methods**: Sixty male Sprague-Dawley rats were induced calcium oxalate crystals by a sodium oxalate diet and divided into six groups, where they drank purified water with or without remineralized varying Ca:Mg (0.5, 3.4, 10, 20, 100). Serum and urine biomarkers of renal function, renal injury, mineral metabolism, bone metabolism, and urine calcium oxalate crystals were detected. Kidneys were isolated for pathological examination. **Results**: Findings showed that remineralization by 0.5 and 3.4 Ca:Mg significantly reduced urinary calcium oxalate crystallization, renal injury, and improved renal function, while extreme ratios (Ca:Mg over 10) showed no benefits. **Conclusions**: These results elucidate the pathophysiological effects of Ca:Mg in drinking water on renal health, particularly emphasizing the protective role of the 0.5 and 3.4 in inhibiting calcium oxalate crystallization and mitigating renal injury. It provides a quantifiable reference for purified water remineralization aimed at stone prevention.

## 1. Introduction

Urinary stone, also known as nephrolithiasis or urolithiasis, is a notable urological disease that is distinguished by the formation of crystals, the majority of which is calcium oxalate [1], in the kidney or urinary tract [2]. It has a high incidence and recurrence and imposes substantial healthcare burdens [3,4]. The influencing factors of urinary stones are diverse, including genetic, environmental, and lifestyle factors. Dietary and drinking patterns have been identified as key factors, especially mineral intake, such as magnesium and citrate, which can treat and prevent urinary stones [5,6].

However, these studies focus on dietary mineral intake or mineral supplements [7]. The influence of water mineral content on nephrolithiasis remains unclear [8]. Minerals in drinking water mainly exist in ionic form, offering high bioavailability and serving as an important source of mineral intake [9,10,11]. Some studies report that stone prevalence is positively associated with hard water consumption [12]. Others suggest an inverse relationship [13,14,15,16,17,18,19]. Drinking water that has high calcium content may reduce the risk of calcium oxalate stones [20]. Mineral water containing calcium and magnesium may be a treatment and prevention measure for calcium oxalate stones [16]. These discrepancies may result from variations in mineral composition, notably the calcium-to-magnesium ratio (Ca:Mg), which may decrease stone incidence [21,22]. Some studies suggest that high calcium in hard water may lead to hypercalciuria, a high risk factor of urinary stone, while high magnesium and bicarbonate can mitigate stone risk [8]. However, others show a positive correlation between tap water Ca:Mg and the incidence of calcium-containing stones [23], and higher magnesium in drinking water may result in urinary stones [24]. Recent research suggests that drinking water with a median Ca:Mg (3–4) exhibits a protective effect against urinary stones [22,25].

However, the aforementioned studies are on the effect of minerals in natural water on urinary stones. The effect of remineralizing low-mineral water, such as purified or desalinated water, on urinary stones remains unclear. Consumption of low-mineral waters cannot provide minerals like magnesium and calcium, potentially disrupting mineral homeostasis [26,27,28,29] and increasing renal burden [30]. Studies show magnesium can inhibit calcium oxalate crystal formation [31], and dietary magnesium intake is negatively correlated with urinary stones [32]. Supplying calcium and magnesium can reduce intestinal oxalate absorption [33]. In addition, magnesium supplementation decreases urinary oxalate excretion while increasing urine magnesium [34,35]. Magnesium ions in urine can break the stability of calcium oxalate crystals via competitively binding with oxalate [36]. Thus, high urine magnesium can prevent the formation and process of calcium oxalate crystals [37,38]. Magnesium supplementation can also increase urine citrate [39,40], which inhibits the formation of calcium oxalate crystals [41,42]. Drinking water is an important source of magnesium intake [43]. Consuming low-mineral water can reduce blood magnesium levels, leading to hypomagnesemia [44,45,46,47,48], which may increase calcium in urine and kidneys [49] and promote urinary stones [50].

Water consumed throughout the day can supply calcium consistently and stably, offering more benefits than medical calcium supplements [51,52], which disrupt calcium homeostasis by delivering large quantities of calcium in a short time [51,53]. Adding magnesium to low-magnesium water can increase urine magnesium in consumers [54]. This raises the following questions: Can adding calcium and magnesium reduce the risk of urinary stones associated with low-mineral water, especially purified water? If feasible, should only calcium be added, or should both calcium and magnesium be supplemented? Is there a specific required ratio for calcium and magnesium addition?

In light of these considerations, this study elucidates the preventive potential of remineralizing purified water with varying Ca:Mg on calcium oxalate crystallization and renal injury utilizing a well-established sodium oxalate-induced nephrolithiasis model in Sprague-Dawley rats (SD rats). Its pathological manifestations are consistent with those observed in patients with calcium oxalate kidney stones, which constitute the largest proportion of all kidney stone cases [55]. This approach enables a controlled evaluation of the specific contributions of calcium and magnesium in drinking water to lithogenic risk and renal health, addressing an understudied aspect of stone prevention relevant to populations consuming low-mineral water and providing a scientific foundation for optimizing drinking water mineral profiles to targeted interventions for urinary stone prevention.

## 2. Materials and Methods

### 2.1. Animals and Reagents

The Institutional Animal Care and Ethics Committee of Army Medical University (Chongqing, China) approved all animal procedures (Ethical number: AMUWEC20198024). All protocols involving animal care and use strictly adhered to the NIH requirements and followed the ARRIVE guidelines.

The calcium sulfate (KA728547, AR, purity ≥ 97%) and magnesium sulfate (KA777213, GR, purity ≥ 99.8%) used in this study were purchased from Shanghai Jieshikai Biotechnology Co., Ltd. (Shanghai, China). They were added to the purified water with different proportions of calcium and magnesium but similar hardness (between the median (144 mg/L) and mean (158 mg/L) hardness of tap water across 314 cities in China) and potential renal acid load (PRAL) (Table 1).

Sixty weaned male SD rats (10–12 week old, weight: 251.6 g ± 17.8 g) were obtained from the Experimental Center of Army Medical University (license: SCXK2012-0009). The rats were randomly divided into six groups (*n* = 10 per group) and had free access to purified water, without (PW group) or with remineralizing by varying Ca:Mg (mass ratios): 0.5 (R0.5 group, close to the minimum Ca:Mg (0.73) of municipal tap water from 314 cities in China [43]), 3.4 (R3.4 group, between the upper limit (3) of yang’s suggestion [22] and the median Ca:Mg (4.23) of tap water across 314 cities in China), 10 (R10 group), 20 (R20 group, close to the maximum Ca:Mg (31.73) of municipal tap water from 314 cities in China), and 100 (R100 group), respectively. The subjects were housed in controlled SPF conditions (five rats a cage) with a 12 h light/12 h dark cycle, 25 ± 1 °C ambient temperature, 50 ± 5%  humidity, and had free access to stone-inducing diet which was prepared by adding 1 g/kg sodium oxalate to the standard maintenance feed (according GB 14924.3-2010 [57]) purchased from Jiangsu Xietong Bio-engineering Co., Ltd. (Nanjing, China). Body weight, water, and diet consumption were recorded every two weeks.

In the 14th week, the animals were fasted, and 24 h urine was collected in metabolic cages for urine analysis. When the calcium oxalate crystals in urine were identified, the subjects were anesthetized by intraperitoneal injection of sodium pentobarbital. It was the only pain management in the experimental protocols because no treatments that could cause pain, suffering, or distress were applied during the housing period. However, three rats died during the anesthesia procedure. The cause might be an excessive injection of sodium pentobarbital (100 mg/kg). After we reduced the anesthetic dose to 60 mg/kg, no further animal deaths were observed. Heart blood was collected and centrifuged at 3000× *g* for 10 min to separate the serum. The six rats (one per group), which were anesthetized with 100 mg/kg sodium pentobarbital, were not included in the serological tests to avoid the impact of different anesthetic dosages. In addition, two serum samples (one from the R10 group and another from the R20) were used up in other tests. The right kidneys were rapidly extracted for histopathology detection. And the left kidneys were rapidly frozen in liquid nitrogen and then stored at −80 °C.

### 2.2. Urine and Serum Analysis

Calcium (Ca), magnesium (Mg), and creatinine (CREA) in serum and urine were detected using the arsenazo III method, the arsenazo I method, and the sarcosine oxidase method, respectively. Serum potassium (K) and sodium (Na) levels were measured via the ion-selective electrode method, phosphorus (P) was determined using the phosphomolybdate method, urea was analyzed by the urease-glutamate dehydrogenase method, uric acid (UA) was detected by the uricase-peroxidase method, and cystatin C was measured by the latex-enhanced immunoturbidimetric method. All these assays were performed using the Beckman AU5800 Automated Chemistry Analyzer (Beckman Coulter, Inc., Brea, CA, USA). Calcium oxalate crystals, urine pH value, and urine specific gravity were detected using the Dirui FUS-2000 automatic urine sediment analyzer (Dirui, Inc., Jilin, China). Above analysis were performed by the clinical laboratory in Xinqiao Hospital (Army Medical University, Chongqing, China), and the testers were unaware of which group the samples came from.

Serum calcium regulator including parathyroid hormone (PTH), calcitonin (CT), vitamin D3, biomarkers of osteoblast and osteoclast activity, bone-specific alkaline phosphatase (BALP) and tartrate-resistant acid phosphatase (TRAP), bone formation and resorption, procollagen type I *N*-terminal propeptide (PINP) and *C*-terminal telopeptide of type I collagen (CTx), renal injury, kidney injury molecule-1 (KIM-1) and neutrophil gelatinase-associated lipocalin (NGAL), and vasopressin (AVP) were detected by ELISA kits (PTH, ml002989; CT, ml002886; vitamin D3, ml028285; BALP, ml003415; TRAP, ml106987; PINP, ml038224; CTx, ml003410; KIM-1, ml003246; NGAL, ml003302; AVP, YJ958110A (Shanghai Enzyme-linked Biotechnology Co., Ltd., Shanghai, China)). The contents of citrate and oxalate in urine were determined using the citrate (BC2155) and oxalate (BC4365) content assay kit (Beijing Solarbio Science & Technology Co., Ltd., Beijing, China), according to the manufacturer’s specifications. The testers were also unaware of which group the samples came from.

### 2.3. Histopathology Analysis

Kidneys were fixed with 4% paraformaldehyde, paraffin-embedded, and sectioned into 4 µm sections. HE staining was performed to observe the pathological changes. Von Kossa and Alizarin red staining were used to detect calcium salt deposition.

### 2.4. Western Blotting

Protein expression of osteopontin (OPN) in the left kidneys was detected by Western blot as previously mentioned [58]. The primary antibody used (OPN, GB112328) and the secondary antibody (HRP-conjugated Goat Anti-Rabbit IgG, GB23303) were from Servicebio (Servicebio Technology Co., Ltd., Wuhan, China). Western blots were visualized by biomolecular imager (Amersham ImageQuant 800, Cytiva (Global Life Sciences Solutions USA LLC), Marlborough, MA, USA), and protein levels were analyzed using Image J software (version 1.5.4r, 1.53t, Wayne Rasband and contributors, National Institutes of Health, Bethesda, MD, USA).

### 2.5. Statistical Methods

All analyses were performed in SPSS (IBM Corp. Released in 2020. IBM SPSS Statistics for Windows, version 27.0. Armonk, NY, USA: IBM Corp). Normal distribution was confirmed in all tests performed by the One-Sample Kolmogorov–Smirnov Test. Group differences were assessed through one-way analyses of variance (ANOVAs) followed by Tukey’s multiple comparisons test. Statistical significance was defined as *p* < 0.05.

## 3. Results

### 3.1. Body Weight, Diet, and Water Consumption After Drinking Different Water

There were no significant differences among the six groups in body weights, diet, and water consumption over 14 weeks (Figure S1A–C).

### 3.2. Comparison of Urinary Calcium Oxalate Crystallization After Drinking Different Water

**Figure 1:**
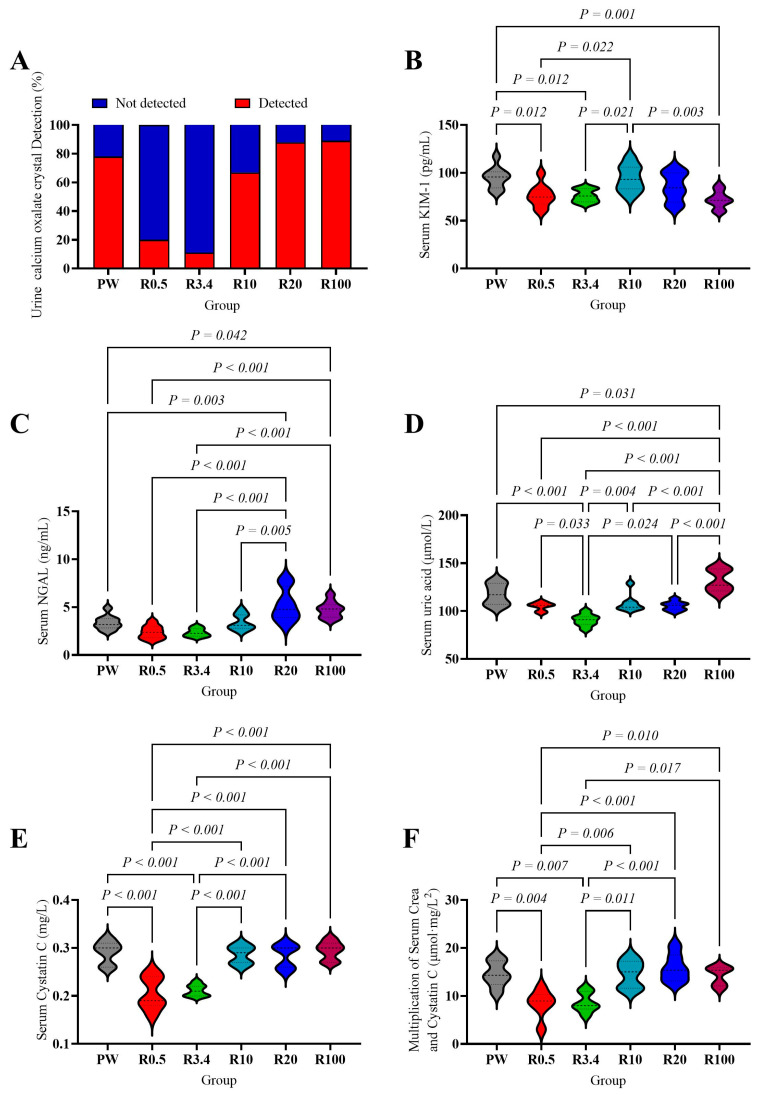
Effects of drinking different waters on urinary calcium oxalate. (**A**) Crystallization and serum biomarkers of kidney injury and renal function (**B**–**F**) of rats. (**A**) Urinary calcium oxalate detestion, *n* = 9 rats/group in PW, R3.4, R10, R100 groups, 10 rats/group in R0.5 group, and 8 rats/group in R20 group. (**B**) Serum KIM-1 concentration. (**C**) Serum NGAL concentration. (**D**) Serum uric acid concentration. (**E**) Serum cystatin C concentration. (**F**) Multiplication of serum CREA and cystatin C. In (**B**–**F**), the values are presented by the violin plot; *n* = 9 rats/group except the R10 and R20 group (*n* = 8 rats/group).

The main function of a stone-inducing diet is to induce calcium oxalate crystals by increasing oxalate excretion. Therefore, we focused on the identification of calcium oxalate crystals in urine. The detection rates of calcium oxalate crystals were as follows: PW group (control) (7/10, 70%), R0.5 group (2/10, 20%), R3.4 group (1/10, 10%), R10 group (7/10, 70%), R20 group (7/9, 77.8%), and R100 group (8/10, 80%). And there are significant differences among groups (*p* < 0.05, Figure 1A).

### 3.3. Comparison of Renal Histopathological Changes After Drinking Different Water

**Figure 2:**
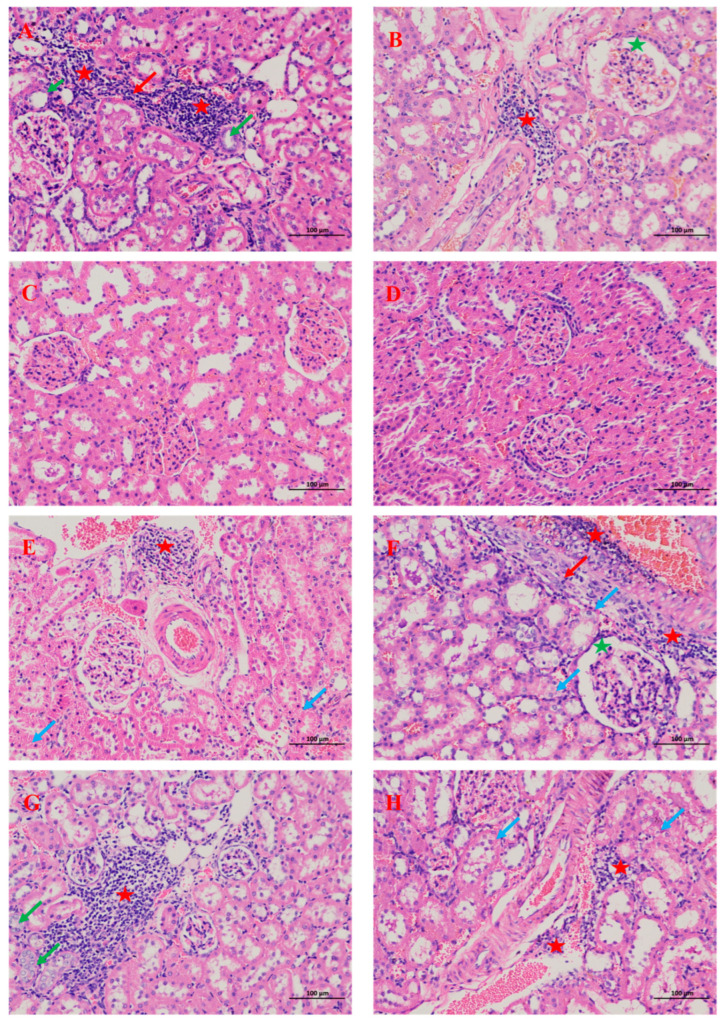
Histopathological changes in the kidneys of rats after consumption of different types of water. (200×), scale bar  =  100 μm. (**A**,**B**) are the kidneys from the rats drinking purified water. (**C**) The kidneys from the rats drinking water remineralized with a calcium–magnesium mass ratio of 0.5. (**D**) The kidneys from the rats drinking water remineralized with a calcium–magnesium mass ratio of 3.4. (**E**) The kidneys from the rats drinking water remineralized with a calcium–magnesium mass ratio of 10. (**F**,**G**) The kidneys from the rats drinking water remineralized with a calcium–magnesium mass ratio of 20. (**H**) The kidneys from the rats drinking water remineralized with a calcium–magnesium mass ratio of 100. Green Arrow: renal tubular atrophy. Red Arrow: connective tissue hyperplasia. Red Star: lymphocyte infiltration. Green Star: Bowman’s capsule dilation. Blue Arrow: renal tubular epithelial edema.

Except for the R0.5 and R3.4 groups, other groups had kidney injury manifestations, including renal tubular epithelial edema and lymphocyte infiltration (Figure 2). The PW group (control) and R20 group also showed renal tubular atrophy, Bowman’s capsule dilation, and connective tissue hyperplasia (Figure 2B,C,F,G). No significant calcium salt deposition was found in any group with Alizarin red and Von Kossa staining.

### 3.4. Comparison of Serological Kidney Injury and Renal Function Biomarkers and Osteopontin Expression in the Kidney After Drinking Different Water

Serum KIM-1 in the PW and R10 groups was significantly higher than in the R0.5, R3.4, and R100 groups (*p* < 0.05, Figure 1B). NGAL in the R20 and R100 groups was significantly higher than in the PW, R0.5, and R3.4 groups; the R20 group’s level was also higher than that of the R10 group (*p* < 0.05, Figure 1C). Serum uric acid content was highest in the R100 group (compared to other groups, *p* < 0.05, Figure 1D) and lowest in the R3.4 group (compared to other groups, *p* < 0.05, Figure 1D). Cystatin C and the multiplication of serum CREA and cystatin C in the R0.5 and R3.4 groups were significantly lower than in the other groups (*p* < 0.05, Figure 1E,F). There were no significant differences in the serum CREA and urea among these groups (*p* > 0.05, Table S1). No significant differences in osteopontin expression in the kidney were observed among the groups (*p* > 0.05, Table S1).

### 3.5. Comparison of Calcium and Magnesium Metabolism After Drinking Different Water

**Figure 3:**
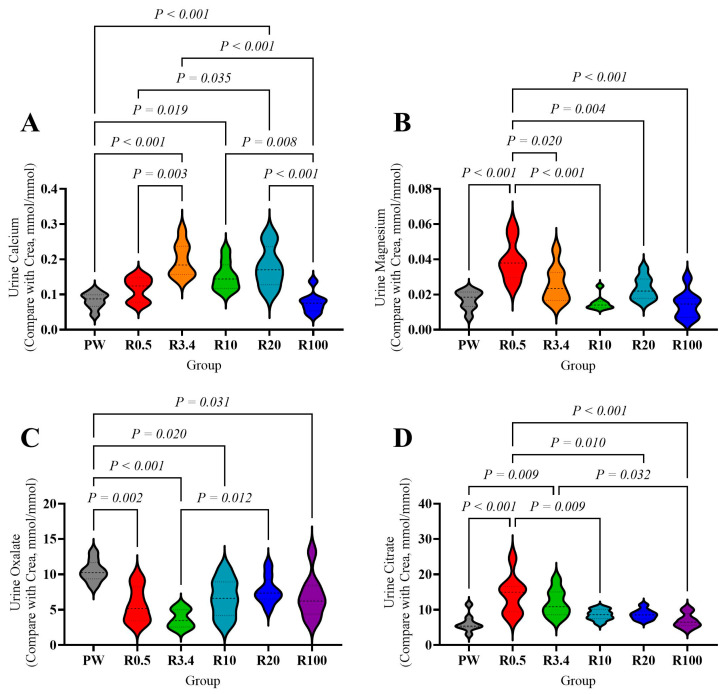
Effects of drinking different water on the urine minerals of rats. (**A**) Urine calcium compared with CREA. (**B**) Urine magnesium compared with CREA. (**C**) Urine oxalate compared with CREA. (**D**) Urine citrate compared with CREA. The values are presented by the violin plot; *n* = 8 rats/group.

There were no significant differences in the serum calcium, magnesium, potassium, sodium, phosphorus, urine pH values and specific gravity among these groups (*p* > 0.05, Tables S1 and S2). After adjustment for urinary creatinine, urinary calcium in the R3.4, R10, and R20 groups was significantly higher than in the PW and R100 groups (*p* < 0.05, Figure 3A). It was also significantly higher in the R3.4 and R20 groups compared to the R0.5 group (*p* < 0.05, Figure 3A). Urinary magnesium in the R0.5 group was significantly higher than in all other groups (*p* < 0.05, Figure 3B). Urinary oxalate in the PW group was significantly higher than in the R0.5, R3.4, R10, and R100 groups, and the R3.4 group’s urinary oxalate was lower than that of the R20 group (*p* < 0.05, Figure 3C). The R0.5 group had the highest urinary citrate content (compared to the PW, R10, R20, and R100 groups, *p* < 0.05, Figure 3D), followed by the R3.4 group (which was higher than the PW and R100 groups, *p* < 0.05, Figure 3D). The exact concentrations of urinary electrolytes, including calcium, magnesium, oxalate, citrate, and creatinine, were also different among groups (*p* < 0.05, Table S2, Figure S2).

### 3.6. Comparison of Calcium Regulatory Hormones, Antidiuretic Hormone, and Biomarkers of Bone Metabolism After Drinking Different Water

**Figure 4:**
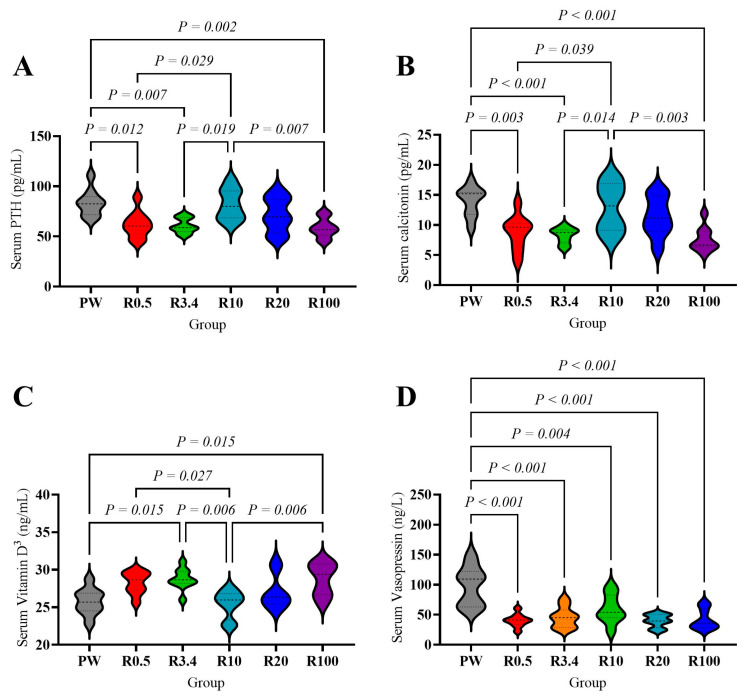
Effects of drinking different waters on serum calcium regulators and antidiuretic hormone of rats. (**A**) Serum parathyroid hormone concentration. (**B**) Serum calcitonin concentration. (**C**) Serum vitamin D3 concentration. (**D**) Serum vasopressin concentration. The values are presented by violin plot; *n* = 9 rats/group except R10 and R20 group (*n* = 8 rats/group).

Serum PTH and calcitonin in the PW and R10 groups were significantly higher than those in the R0.5, R3.4, and R100 groups (*p* < 0.05, Figure 4A,B). Vitamin D3 in the PW group was lower than that in the R3.4 and R100 groups (*p* < 0.05, Figure 4C), being significantly lower in the R10 group compared to the R0.5 group (*p* < 0.05, Figure 4C). Vasopressin was significantly higher in the PW group compared to other groups (*p* < 0.05, Figure 4D).

**Figure 5:**
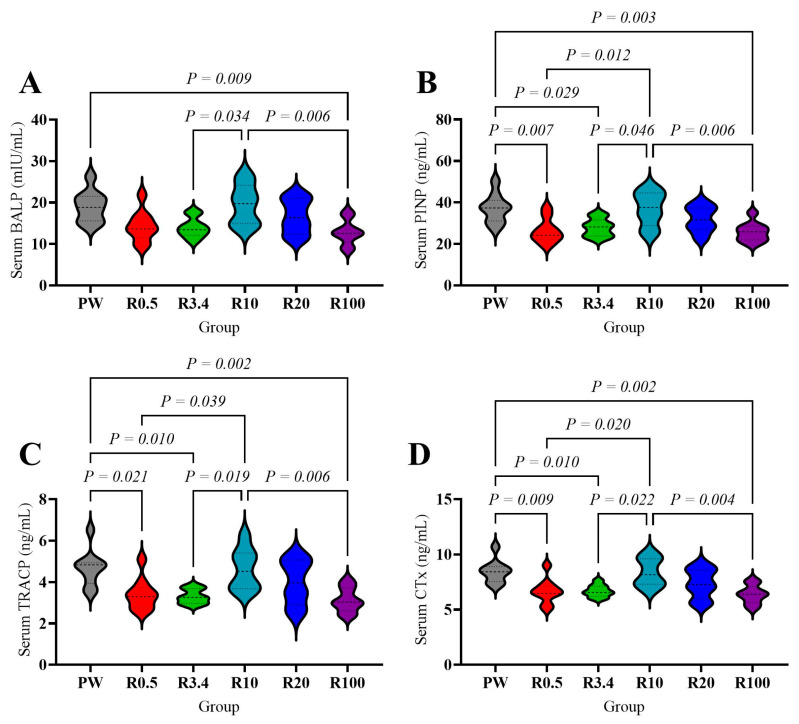
Effects of drinking different waters on serum biomarkers of bone metabolism of rats. (**A**) Serum bone-specific alkaline phosphatase concentration. (**B**) Serum procollagen type I *N*-terminal propeptide concentration. (**C**) Serum tartrate-resistant acid phosphatase concentration. (**D**) Serum *C*-terminal telopeptide of type I collagen concentration. The values are presented by violin plot; *n* = 9 rats/group except R10 and R20 group (*n* = 8 rats/group).

Serum BALP was significantly higher in the PW and R10 groups than in the R100 group (*p* < 0.05, Figure 5A) and higher in the R10 group compared to the R3.4 group (*p* < 0.05, Figure 5A). The serum PINP, TRAP, and CTx were significantly higher in the PW and R10 groups compared to the R0.5, R3.4, and R100 groups (*p* < 0.05, Figure 5B–D).

## 4. Discussion

This study evaluates the effects of remineralizing purified water with varying Ca:Mg on urinary calcium oxalate crystallization, renal injury and function, and mineral and bone metabolism. Our findings reveal that remineralization can mitigate urine calcium oxalate crystal and renal injury induced by low-mineral water consumption. Notably, the protective ability was dependent on the proportion of calcium and magnesium in the remineralization process. These results provide critical insights into the interplay between minerals in drinking water and nephrolithiasis incidence, providing evidence for developing novel interventions targeting water remineralization to urinary stones.

Various urinary parameters are employed to assess urinary stone risk, among which the presence of calcium oxalate crystals in urine is the most direct indicator [59]. The observed reduction in urinary calcium oxalate crystallization following remineralization with specific calcium-to-magnesium ratios underscores the critical role of mineral balance in modulating nephrolithiasis risk [60]. Moreover, the biphasic response was observed where higher calcium proportions (Ca:Mg over 10) reversed the protective effect, echoing epidemiological data indicating that elevated calcium-to-magnesium ratios in drinking water correlate positively with stone formation risk. Thus, controlled remineralization optimizes the inhibitory effects of magnesium while avoiding calcium overload, mitigating nephrolithiasis. Beyond urinary crystals, renal pathological changes showed a similar trend. Although no calcium deposits were observed, rats in the purified water group exhibited histopathological changes in kidney injury. They were alleviated after remineralization (R0.5 and R3.4 groups). However, as the calcium proportion in remineralization increased (Ca:Mg over 10), they reappeared. KIM-1, a transmembrane glycoprotein upregulated in proximal tubular injury, serves as an early and sensitive indicator of renal tubular damage [61], while NGAL reflects tubular epithelium stress and inflammatory activation, identifying individuals at risk of rapid chronic kidney disease progression [62]. This study finds that moderate remineralization reduces KIM-1, suggesting diminished tubular epithelial injury. Conversely, excessive calcium addition correlated with increased KIM-1 (R10 group) and NGAL (R20 and R100 group), indicating persistent renal injury and inflammation.

Renal injury often accompanies changes in renal function. Uric acid, the end product of purine metabolism in humans, is primarily excreted through the kidneys and can reflect renal metabolic function [63]. Serum cystatin C, a low-molecular-weight protein steadily synthesized by nucleated cells, is almost entirely filtered by the glomeruli and broken down in the proximal tubules after release into the bloodstream. Its concentration is mainly determined by glomerular filtration rate and is less influenced by age, sex, or muscle mass, making it a sensitive indicator of renal function [64]. The multiplication of serum creatinine and cystatin C is based on the CKD-EPI creatinine–cystatin C equation, which reduces interference from extra-renal factors and is particularly suitable for assessing early renal impairment [65]. All of them decrease in the R0.5 and R3.4 groups and rise again after another addition of calcium (Ca:Mg over 10), indicating improved glomerular filtration function after remineralization and relapse after excessive calcium addition.

Urinary crystal formation and renal injury can result from variations in mineral excretion. Our previous research found that consuming purified water disrupts calcium and magnesium metabolism, especially calcium and magnesium in blood [30,66,67,68]. However, serum potassium, sodium, calcium, magnesium, and phosphorus showed no significant differences among the groups in this study. But urinary calcium, magnesium, and citrate increased significantly, while oxalate decreased (urinary calcium: R3.4, R10, R20 groups; urinary magnesium: R0.5 group; urinary citrate: R0.5 and R3.4 groups; urinary oxalate: R3.4, R10, R100 groups). These benefits diminished as the calcium proportion increased (calcium: over 100; magnesium: over 3.4; citrate: over 10). The elevation of urinary magnesium and citrate alongside decreased oxalate excretion under balanced remineralization supports magnesium’s role in enhancing citrate-mediated inhibition of crystallization, as citrate complexes calcium, reducing supersaturation [41]. Furthermore, the dynamic changes observed in calcium-regulating hormones such as PTH, vitamin D3, and calcitonin suggest feedback adjustments to maintain mineral homeostasis, consistent with known interactions where magnesium modulates PTH secretion and vitamin D3 metabolism. Compared to purified water, remineralization increased vitamin D3 and decreased calcitonin (R0.5 and R3.4 groups). As the calcium addition increased, vitamin D3 first decreased, then increased, while calcitonin first increased, then decreased (R10 vs. R100 group), potentially explaining the elevated urinary calcium in R10 and R20 groups and the reduction in the R100 group. PTH decreased after remineralization (R0.5 and R3.4) and fluctuated with increasing calcium addition (Ca:Mg over 10). In the R3.4 group, reduced PTH combined with lower urinary magnesium promoted calcium excretion, leading to increased urinary calcium. However, the R3.4 group also had low urinary oxalate, high citrate, and magnesium, which may explain its low crystal formation and renal injury [42]. The inverse relationship between PTH and vitamin D_3_ indicates that vitamin D_3_ exerts predominant negative feedback on PTH secretion.

These changes in mineral metabolism and calcium-regulating factors also affected bone metabolism. The biomarker of bone formation (PINP) and resorption (CTx), and the osteoblast and osteoclast activity (BALP and TRAP), decreased after remineralization. The suppression of both bone formation and resorption at Ca:Mg (0.5 and 3.4) may reflect a normalization of bone modeling and remodeling disrupted by mineral imbalance [30,66,67], preventing excessive calcium release from bone, which will exacerbate nephrolithiasis. These markers increased again with higher calcium addition (Ca:Mg over 10), which may result from elevated calcium intake. Notably, when calcium addition was much higher (Ca:Mg over 100), both bone formation and resorption decreased again. Considering the changes in calcium-regulating hormones, mineral metabolism, renal injury, and renal function in the R100 group, excessive calcium supplement without magnesium may dysregulate the balance of calcium and magnesium, then induce calcium overload and disturb calcium-regulating hormones, which promote urinary stone, renal injury, and aberrant bone metabolism. Thus, the proportion of calcium and magnesium should be considered in the remineralization process.

Besides mineral metabolism, remineralization also affects the antidiuretic hormone, vasopressin, which promotes water reabsorption in the kidneys to concentrate urine [69]. Remineralization of purified water reduces the serum vasopressin, independent of the calcium-to-magnesium ratio. This suggests that remineralization can promote diuresis, reduce urine concentration, and lower the risk of kidney stones [70]. This effect was independent of the calcium-to-magnesium ratio employed during remineralization. On the other hand, it also indicates that the different effects of remineralization with different calcium-to-magnesium ratios on renal function and renal crystal formation are not related to urine concentration.

This study has several limitations. First, to avoid over-induction of urinary stones that could mask the effects of different Ca:Mg [71], we reduced the sodium oxalate content in the stone-inducing diet. However, this resulted in no observable calcium oxalate crystals or deposits in pathological sections, affecting the reliability of our outcomes. Second, consistent with our earlier research, the impact of water minerals on mineral metabolism is subtle, systemic, and whole-body. This study also did not identify key factors or core mechanisms, limiting the scientific interpretation of the results. Third, the experimental duration was relatively short, preventing assessment of the long-term effects of drinking remineralized water. Fourth, due to the small number of groups, the calcium–magnesium ratios across different groups show a large disparity, especially in the effect-reducing group (Ca:Mg over 10). We were unable to establish a dose–response relationship between crystallization and the calcium–magnesium ratio. Additionally, low-mineral water like purified or desalinated water requires acidification before remineralization to facilitate the dissolution of minerals [72]. Given that sulfuric acid is currently the mainstream acidification process, we added calcium sulfate and magnesium sulfate without incorporating bicarbonate. Bicarbonate in drinking water helps maintain acid–base balance, reduces the risk of metabolic acidosis [73], and lowers renal acid load and calcium excretion [30]. The lack of sufficient bicarbonate may explain why remineralization in this study did not reduce renal calcium metabolism but instead increased calcium excretion—possibly as a compensation to balance renal acid load and mitigate kidney injury [30].

## 5. Conclusions

This study reveals that the proportion of calcium and magnesium in remineralized water influences calcium oxalate crystallization, renal injury, and renal function. Our findings indicate that a Ca:Mg ratio between 0.5 and 3.4 in remineralization significantly reduces urinary calcium oxalate crystallization and mitigates renal injury markers. These insights not only enhance our understanding of the role of mineral balance in urinary stones but also highlight the potential of remineralizing purified water as an intervention for calcium oxalate stones. Future research should concentrate on the underlying mechanisms.

## Figures and Tables

**Table 1:** Minerals in the remineralized water.

Group	CaSO_4_ (mg/L)	MgSO_4_ (mg/L)	Ca (mg/L)	Mg (mg/L)	Sulfate (mg/L)	Ca:Mg	PRAL ^1^ (mEq/L)	Hardness (CaCO, mg/L)
PW	0	0	0	0	0.0		0.0	0.0
R0.5	48	139	14.1	28.1	144.8	0.5	1.2	150.8
R3.4	134	58	39.4	11.7	140.6	3.4	1.2	146.5
R10	178	26	52.4	5.3	146.4	9.9	1.3	152.6
R20	192	14	56.5	2.8	146.4	20.2	1.3	152.5
R100	206	3	60.6	0.6	147.5	101.0	1.4	153.7

^1^ PRAL of water was calculated using the formula from Wynn [56]: PRAL (mEq/L) = 0.0146 × sulfates (mg/L) + 0.027 × chlorides (mg/L) + 0.037 × phosphorus (mg/L) − 0.021 × potassium (mg/L) − 0.026 × magnesium (mg/L) − 0.0413 × sodium (mg/L) − 0.013 × calcium (mg/L).

## Data Availability

The original contributions presented in the study are included in the article/Supplementary Material, further inquiries can be directed to the corresponding authors.

## Supplementary Materials

The following supporting information can be downloaded at: https://www.mdpi.com/article/10.3390/nu18050792/s1, Figure S1. Effects of drinking different waters on body weight (A), diet (B), and (C) water consumption. Figure S2. Effects of drinking different water on the urine minerals of rats. Table S1. Effects of drinking different waters on serum minerals, biomarkers of renal function, and renal osteopontin expression of rats (mean ± standard error, *n* = 8 per group). Table S2. Effects of drinking different waters on urine minerals, specific gravity, and pH of rats (mean ± standard error).

## Abbreviations

The following abbreviations are used in this manuscript:

Ca:Mg	Calcium-to-magnesium ratios
SD rats	Sprague-Dawley rats
PRAL	Potential renal acid load
Na	Sodium
K	Potassium
Ca	Calcium
Mg	Magnesium
P	Phosphorus
CREA	Creatinine
UA	Uric acid
PTH	Parathyroid hormone
CT	Calcitonin
BALP	Bone-specific alkaline phosphatase
TRAP	Tartrate-resistant acid phosphatase
PINP	Procollagen type i *N*-terminal propeptide
CTx	*C*-terminal telopeptide of type i collagen
KIM-1	Kidney injury molecule-1
NGAL	Neutrophil gelatinase-associated lipocalin
AVP	Vasopressin
OPN	Osteopontin
ANOVAs	One-way analyses of variance
