# Supplementary material for: Impact of Calcium–Magnesium Ratio in Purified Water Remineralization on Calcium Oxalate Crystal Formation and Renal Injury
Source: Nutrients. 2026 Feb 27;18(5):792. doi: 10.3390/nu18050792 (PMC12986501; doi:10.3390/nu18050792)
Supplement: Supplementary file 1 [file nutrients-18-00792-s001.zip › nutrients-4130265-supplementary.pdf]

Impact of Calcium–Magnesium Ratio in Purified Water Remineralization on Calcium Oxalate Crystal Formation and Renal Injury

Yingbin Zhang [1,†], Jiaohua Luo [2,†], Yao Tan [2], Zhiqiang Wang [3], Kun Qian [3], Weiyan Chen [2], Ke Cui [2], Ji-An Chen [1,*] and Yujing Huang [2,*]

[1] Department of Health Education, College of Preventive Medicine, Army Medical University, Chongqing 400038, China; yingbinzhang@tmmu.edu.cn
[2] Department of Environmental Hygiene, College of Preventive Medicine, Army Medical University, Chongqing 400038, China; ljh978@tmmu.edu.cn (J.L.); xiaoyue7122@tmmu.edu.cn (Y.T.); weiyanchen@tmmu.edu.cn (W.C.); cuike@tmmu.edu.cn (K.C.)
[3] Department of Environmental Science and Engineering, School of Environmental Studies, China University of Geosciences, Wuhan 430074, China; cugwangzq@126.com (Z.W.); qkwell2046@cug.edu.cn (K.Q.)
* Correspondence: cjatmmu@hotmail.com (J.-A.C.); huangyujing@tmmu.edu.cn (Y.H.)
† These authors contributed equally to this work.

Supplementary Figures

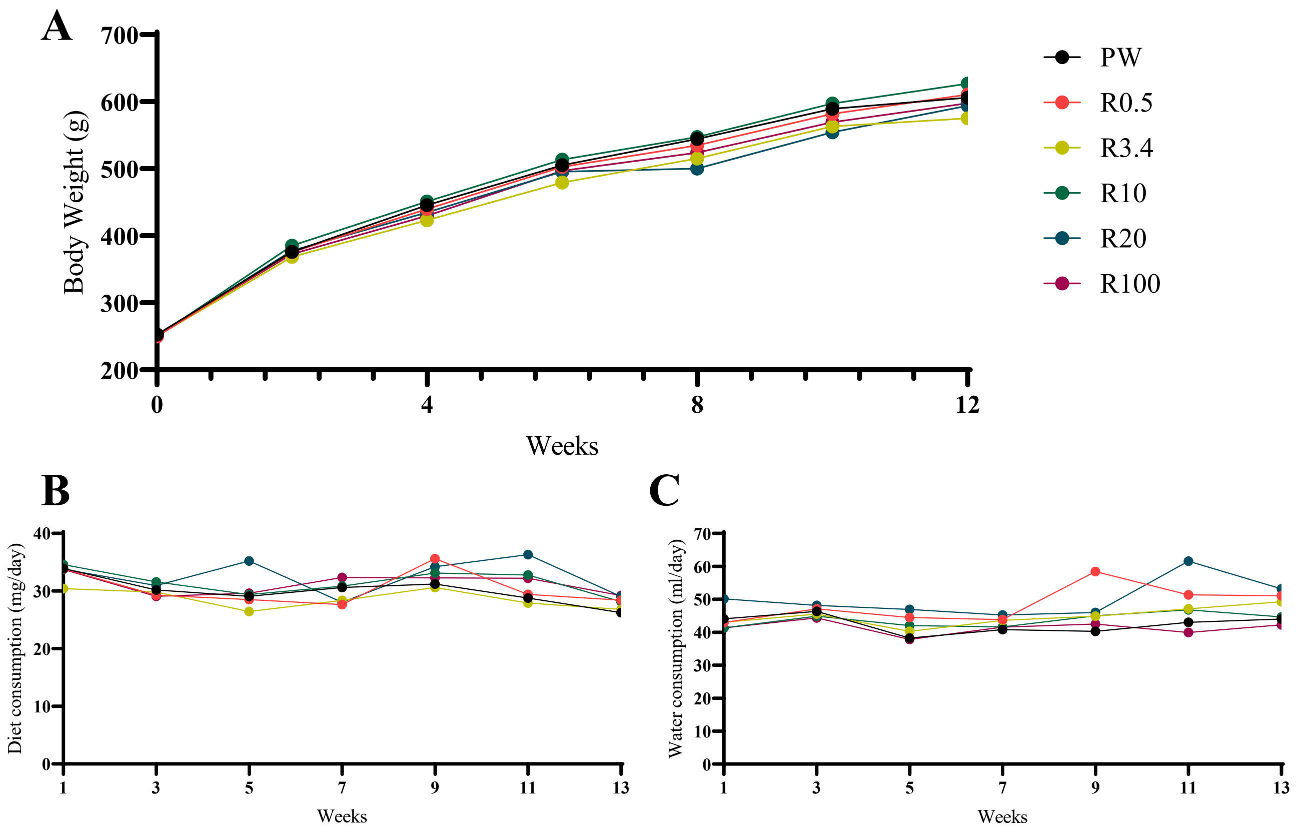

Figure S1. Effects of drinking different waters on body weight (A), diet (B), and water (C) consumption.

(A) body weight. (B) diet consumption. (C) water consumption. The values are presented by the violin plot; n = 10 rats/group. PW group: The rats drink purified water. R0.5 group: The rats drink water remineralized with a calcium-magnesium mass ratio of 0.5. R3.4 group: The rats drink water remineralized with a calcium-magnesium mass ratio of 3.4. R10 group: The rats drink water remineralized with a calcium-magnesium mass ratio of 10. R20 group: The rats drink water

remineralized with a calcium-magnesium mass ratio of 20. R100 group: The rats drink water remineralized with a calcium-magnesium mass ratio of 100.

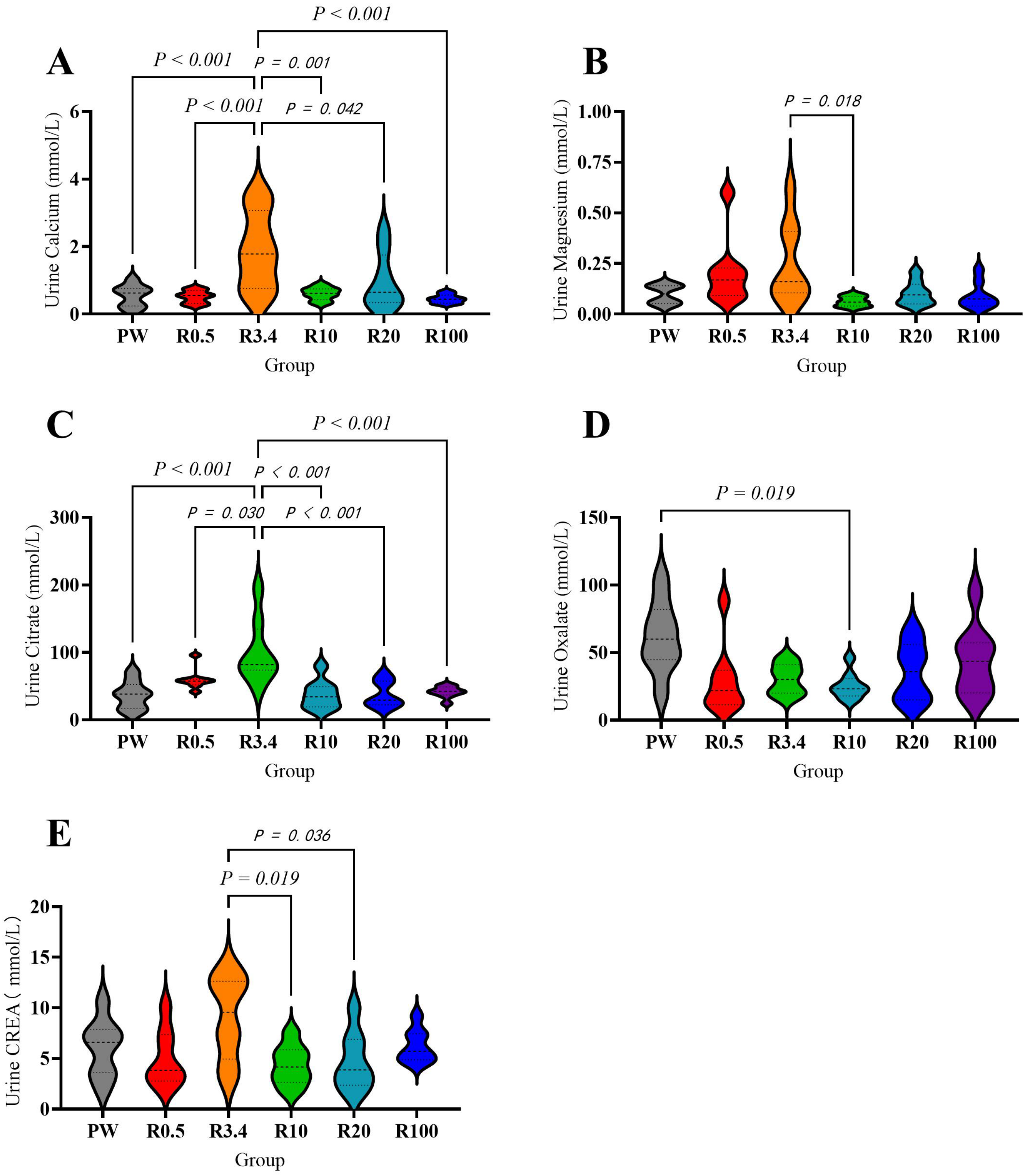

Figure S2. Effects of drinking different water on the urine minerals of rats. (A) urine calcium. (B) urine magnesium. (C) urine oxalate. (D) urine citrate. (E) urine CREA. The values are presented by the violin plot; n = 8 rats/group.

Supplementary Tables

Table S1. Effects of drinking different waters on serum minerals, biomarkers of renal function, and renal osteopontin expression of rats.

Group	Adjusted Ca[1] (mmol/L)	Mg (mmol/L)	Na (mmol/L)	K (mmol/L)	P (mmol/L)	UREA (mmol/L)	CREA (μmol/L)	Osteopontin[2]
PW	3.26±0.28	1.35±0.24	158.41±25.14	9.23±2.03	4.37±0.80	6.90±1.05	47.00±9.97	1.010±0.021
R0.5	3.35±0.20	1.44±0.21	171.25±16.95	9.47±1.94	3.76±0.45	7.54±0.98	44.29±13.86	0.993±0.029
R3.4	3.12±0.17	1.31±0.13	161.42±15.77	8.81±1.44	3.45±0.37	6.36±0.85	41.43±7.72	1.004±0.020
R10	3.32±0.24	1.24±0.23	168.09±20.91	9.03±1.80	3.68±0.69	6.43±0.49	45.71±8.62	1.032±0.018
R20	3.48±0.18	1.38±0.13	176.24±17.96	9.72±1.12	3.86±0.52	7.46±1.08	53.29±11.37	1.042±0.026
R100	3.46±0.23	1.26±0.14	171.19±17.57	9.25±2.16	4.23±0.92	7.41±1.02	51.86±12.99	0.978±0.020

mean ± standard error, n = 9 rats/group except the R10 and R20 group (n=8 rats/group)

[1]Adjusted by albumin following the formula: Adjusted calcium (mmol/L)=serum calcium (mmol/L)+0.02×(40-serum albumin(g/L))

[2] Compared with the duplicate sample, which was run on each gel as a reference, n=8.

Table S2. Effects of drinking different waters on Urine minerals, specific gravity, and pH of rats ().

Group	Ca (mmol/L) (n=8)	Mg (mmol/L) (n=8)	P (mmol/L) (n=8)	Oxlate (mmol/L) (n=8)	Citrate (mmol/L) (n=8)	CREA (mmol/L) (n=8)	Urine Specific gravity[1]	Urine pH value[1]
PW	0.55±0.11	0.098±0.016	8.77±1.17	61.47±9.38	36.81±7.27	6.06±1.01	1.009±0.002	7.33±0.25
R0.5	0.51±0.07	0.206±0.060	8.82±1.27	29.58±9.16	60.01±5.60	4.99±1.00	1.012±0.002	6.30±0.31
R3.4	1.90±0.40	0.256±0.070	10.92±0.52	30.43±4.01	100.38±16.76	9.09±1.45	1.013±0.003	6.39±0.31
R10	0.60±0.07	0.063±0.009	9.06±0.56	25.48±3.52	37.82±7.55	4.33±0.70	1.011±0.002	6.89±0.34
R20	0.97±0.30	0.103±0.021	8.71±1.01	35.96±7.62	37.95±6.88	4.69±1.02	1.013±0.002	6.56±0.36
R100	0.45±0.05	0.091±0.023	11.52±0.36	44.60±9.20	41.33±3.02	6.20±0.58	1.013±0.003	6.39±0.29

mean ± standard error

[1]n=9 rats/group in PW, R3.4, R10, R100 groups, 10 rats/group in R0.5 group, and 8 rats/group in R20 group.
